# Pu-Erh Tea Relaxes the Thoracic Aorta of Rats by Reducing Intracellular Calcium

**DOI:** 10.3389/fphar.2019.01430

**Published:** 2019-11-28

**Authors:** Dan Luo, Xuejiao Chen, Xu Zhu, Shuang Liu, Jie Li, Jianping Xu, Jinhua Zhao, Xu Ji

**Affiliations:** ^1^State Key Laboratory of Phytochemistry and Plant Resources in West China, Kunming Institute of Botany, Chinese Academy of Sciences, Kunming, China; ^2^University of Chinese Academy of Sciences, Beijing, China; ^3^Key Laboratory of Medicinal Chemistry for Natural Resource, Ministry of Education and Yunnan Province, School of Chemical Science and Technology, Yunnan University, Kunming, China

**Keywords:** endothelium-independent vasodilation, hypertension, pu-erh tea, tea components, theabrownins

## Abstract

Previous studies suggested that pu-erh tea aqueous extract could lower blood pressure and ameliorate hypertension symptoms. However, the antihypertension mechanisms of pu-erh tea remain unclear. In this work, the direct effects of pu-erh tea on vessels and cells were investigated by detecting isometric tension and intracellular calcium ([Ca^2+^]_i_), respectively. Additionally, to identify the main active components, the aqueous extract of pu-erh was separated by organic solvents to obtain various fractions, and the effects of these fractions on arteries were assessed. The results showed that pu-erh aqueous extract vasodilated rat thoracic aortas preconstricted by phenylephrine or KCl. These vasodilation effects were not significantly affected by the removal of the endothelium or by preincubation with potassium channel blockers (tetraethylammonium, glibenclamide, aminopyridine, or barium chloride). Moreover, pu-erh aqueous extract could reduce the vessel contractibility induced by CaCl_2_ and phenylephrine under KCl-depolarizing or Ca^2+^-free buffer conditions, respectively. Furthermore, pu-erh aqueous extract attenuated the KCl-induced increase in [Ca^2+^]_i_ in cultured rat aortic smooth muscle A7r5 cells. In addition, the chloroform precipitate of pu-erh aqueous extract produced the most potent vasodilation. Theabrownins (the characteristic components of pu-erh tea) accounted for 41.91 ± 1.09 % of the chloroform precipitate and vasodilated arteries in an endothelium-independent manner. In addition, the vasodilation effect of caffeine was verified. In conclusion, theabrownins and caffeine should be the two main active components in pu-erh tea. Pu-erh aqueous extract vasodilated arteries in an endothelium-independent manner, which might partly be attributed to the decrease in extracellular Ca^2+^ influx. Moreover, our study provided data on the potential mechanism of the hypotensive actions of pu-erh tea, which might improve our understanding of the effect of pu-erh tea on the prevention and treatment of hypertension.

## Introduction

Hypertension is a major public health problem with a high morbidity rate and is a common risk factor for cerebrovascular, cardiovascular, and renal disease. One of the pathological mechanisms underlying hypertension is vascular endothelial dysfunction (Taddei et al., 1993; Luscher, 1994). Moreover, hypertension is related to pathological changes in vascular smooth muscle (Wellman et al., 2001; Cox, 2002; Kohler et al., 2010). Therefore, the regulation of the endothelium and vascular smooth muscle may be an important therapeutic strategy for hypertension. Studies have suggested that the chronic consumption of tea could lower blood pressure and reduce cardiovascular risk (Hodgson et al., 2003; Grassi et al., 2008; Serban et al., 2015; Yarmolinsky et al., 2015).

Tea is a widely consumed beverage and has been considered a crude medicine for more than 4,000 years (Cheng, 2004). Based on the degree of fermentation, tea can be classified into four major categories: nonfermented green tea, partially fermented oolong tea, fully fermented black tea, and post-fermented pu-erh tea (Xie et al., 2017). The main components of nonfermented green tea are catechins, including epicatechin (EC), epigallocatechin (EGC), epicatechin-3-gallate (ECG), and epigallocatechin-3-gallate (EGCG) (Lorenz et al., 2009). During the fermentation process, these monomeric catechins are converted to polymeric complexes, known as theaflavins (TFs), thearubigins (TRs), and theabrownins (TBs) (Zhang et al., 2011).

Numerous epidemiological studies have indicated that the dietary consumption of fruit, vegetables and beverages rich in polyphenols is associated with cardiovascular protection, which may be due to the regulatory effect of polyphenols on endothelial function (Ghosh and Scheepens, 2009). Research on isolated blood vessels, animal models, and clinical studies have shown that green tea, black tea, and their polyphenols exert vascular protective effects (Fitzpatrick et al., 1995; Potenza et al., 2007; Ghosh and Scheepens, 2009).

Pu-erh tea is made from the leaves and buds of the tea plant [*Camellia sinensis* var. *assamica* (L.) O. Kuntze; Theaceae]. Pu-erh tea is a traditional beverage in China and has become popular in many countries in Asia due to its health benefit. According to the *Compendium of Materia Medica* by Li Shizhen of the Ming Dynasty, pu-erh tea could expel wind-evil, clear away heat, and aid in weight loss (Wang et al., 2012). Additionally, in modern medicine, pu-erh tea aqueous extract could lower blood pressure (Li et al., 2012) and could synergize with the antihypertensive drug nifedipine in spontaneous hypertension rats (Li et al., 2015). Moreover, many components in pu-erh tea, including EGCG (Potenza et al., 2007; Al Disi et al., 2015; Anwar et al., 2016), EC (Galleano et al., 2013; Jackson et al., 2018), gallic acid (GA) (Jin et al., 2017; Jin et al., 2018a), caffeine (CAF) (Conde et al., 2012; Yeh et al., 2014), etc., could lower blood pressure and ameliorate hypertensive symptoms in different animal models of hypertension. However, the antihypertensive mechanisms of pu-erh tea and its active components are unclear. In this work, the vasodilative activities of pu-erh tea aqueous extract and its main components were investigated *ex vivo*, and the underlying mechanism was explored. The purpose of this study was to better understand the effect of pu-erh tea in delaying the development of hypertension.

## Materials and Methods

### Chemicals and Drugs

Pu-erh tea aqueous extract and CAF were kindly provided by the Pu-erh Tea Research Institute. Pu-erh tea was deposited in the Chinese Virtual Herbarium in Kunming, Yunnan Province (voucher number: KUN 56076). TBs were kindly provided by Yunan Pu-erh Tea Trade Co. Ltd., China. 4-Aminopyrimide (4-AP), tetraethylammonium (TEA), glibenclamide (GLi), barium chloride (BaCl_2_), phenylephrine (PE), acetylcholine chloride (Ach), and EGCG were purchased from Sigma-Aldrich Inc. Catechin (C), EC, ECG, EGC, and GA were purchased from Dalian Meilun Biotechnology Co., Ltd., China. The purities of these compounds were all above 98 %.

### Preparation of Pu-Erh Tea Extracts

Pu-erh aqueous extract was prepared as described previously (Xie et al., 2017). To screen the main active components, pu-erh aqueous extract was treated with 100 % ethanol (20-fold v/w pu-erh aqueous extract) at 60 °C for 48 h. To reduce the content of CAF, the ethanol precipitate of pu-erh aqueous extract was further treated with chloroform (20-fold v/w ethanol precipitate) at 65 °C for 24 h by a Soxhlet extractor. The extracts and precipitates were concentrated by a rotary evaporator (Buchi, Switzerland) and were then dried. The ethanol extract, ethanol precipitate, chloroform extract, and chloroform precipitate yields were 7.02 %, 50.02 %, 47.50 %, and 0.13 %, respectively.

### Animals

Adult male Wistar rats (250 ∼ 300 g) were kept in an animal room with a constant temperature of 22 ± 2 °C and a humidity of 60 ± 5 % and were allowed free access to food and water. The animals were raised in an SPF environment in a licensed laboratory animal facility (accreditation no. SYXK K2013-0004). All animals were treated in compliance with the National Institutes of Health Guide for the Care and Use of Laboratory Animals (NIH publication no. 8023, revised 1978). All experimental procedures were preapproved by the Experimental Animal Ethics Committee of Kunming Institute of Botany, Chinese Academy of Sciences.

### Quantification of Major Chemical Components

The contents of CAF, GA, and tea catechins (C, EC, ECG, EGC, and EGCG) were determined using HPLC (Agilent 1100, USA) based on the method reported previously (Seo et al., 2015). The contents of TFs, TRs, and TBs were determined by a system approach according to previous research (Wang et al., 2011).

### Preparation of Aortic Rings

Isometric tension was assayed as described previously (Ji et al., 2013a; Ji et al., 2013b). Rats were anesthetized with sodium pentobarbital (60 mg/kg, *i.p.*) and sacrificed. The thoracic arteries were carefully removed, and the connective tissue and fat were removed. Finally, the arteries were cut into rings 2-3 mm in length. The endothelium was denuded by gently rubbing the surface of the vessel lumen. The rings were mounted on two stainless steel hooks and immersed in a 5 ml chamber bath containing Krebs solution (composition in mmol/L: NaCl, 120; KCl, 4.7; NaHCO_3_, 25; KH_2_PO_4_, 1.2; MgSO_4_, 1.2; CaCl_2_, 2.5; glucose, 11; pH 7.35) at 37 °C and continuously bubbled with 95 % O_2_ and 5 % CO_2_. The aortic rings were equilibrated for 1 h at 1.5 g of resting tension, and during this equilibrium period, the buffer was changed every 15 min. The integrity of the endothelium was verified with acetylcholine (10 µmol/L) in rings precontracted with PE (1 µmol/L); rings with more than 80 % relaxation were considered to have endothelial integrity, whereas those with less than 10 % relaxation were regarded to have endothelial denudation. The responses of aortic rings were detected using a RM6240 multichannel physiological signal acquisition system (Chengdu Instrument, China).

### Effect of Pu-Erh Aqueous Extract on Vascular Tension

After 60 min of equilibration under baseline tension, the aortic rings were treated with increasing concentrations of pu-erh aqueous extract (0.1–10 mg/ml) at 10 min intervals.

Pu-erh aqueous extract (0.1–10 mg/ml) was added cumulatively at 10 min intervals to the Krebs solution, in which the aortic rings were precontracted with PE (1 µmol/L) or KCl (60 mmol/L). The role of the endothelium in the vasodilation effect of pu-erh tea was investigated by removing the endothelium. To elucidate the role of K^+^ channels in the vasodilation effect of pu-erh aqueous extract, the rings were incubated with various K^+^ channel blockers, including 5 mmol/L TEA (a nonselective inhibitor of Ca^2+^-activated K^+^ channels), 10 µmol/L GLi (an inhibitor of K_ATP_ channels), 1 mmol/L 4-AP (an inhibitor of K_V_ channels), and 1 mmol/L BaCl_2_ (an inhibitor of K_ir_ channels), for 20 min before treatment with PE. The vasodilation effect of pu-erh aqueous extract was calculated as the percentage of PE-induced or KCl-induced vasoconstriction.

To assess the effect of pu-erh aqueous extract on the vasoconstriction induced by extracellular Ca^2+^ influx, endothelium-denuded aortic rings in Ca^2+^-free buffer were pretreated with pu-erh aqueous extract for 15 min and then treated with KCl (60 mmol/L), followed by cumulative concentrations of CaCl_2_ (0.1–10 mol/L). The vasoconstriction effect was expressed as the percentage of the maximal vasoconstriction induced by KCl (60 mmol/L) in normal Krebs buffer. To elucidate the effect of pu-erh aqueous extract on the contractile response of aortic vessels induced by PE in Ca^2+^-free buffer, PE-induced vasoconstriction was carried out before or after treatment with pu-erh tea aqueous extract for 15 min as described previously (Pantan et al., 2014). The inhibitory effect of pu-erh tea aqueous extract was expressed as the second contraction (Con2)/the first contraction (Con1).

### Effect of Various Fractions of Pu-Erh Aqueous Extract on Vascular Tension

Based on the concentration of pu-erh aqueous extract required for the maximum vasodilation effect and on the yield of the various extracts, the concentrations of the ethanol extract, ethanol precipitate, and chloroform extract were calculated. These fractions from pu-erh aqueous extract were used to treat endothelium-intact arteries precontracted with PE or KCl as described above. The maximum vasodilation ratio of various extracts within 30 min was calculated.

### Effect of Major Chemical Components in Pu-Erh Aqueous Extract on Vascular Tension

Based on the concentration of pu-erh aqueous extract required for the maximum vasodilation effect and on the content of major chemical components, the concentrations of C, EC, ECG, EGC, EGCG, GA, and CAF were calculated and applied to study their impact on vascular tension as described above.

### Effect of TBs on Vascular Tension

Arteries with or without endothelium were precontracted with KCl (60 mmol/L) and were then treated with TBs at increasing concentrations (0.5–16 mg/ml) at 10 min intervals.

### Measurement of [Ca^2+^]_i_

To determine whether pu-erh aqueous extract affected the Ca^2+^ influx induced by KCl-mediated depolarization, we measured [Ca^2+^]_i_ in cultured rat aortic smooth muscle A7r5 cells (Cell Resource Center, Shanghai Academy of Life Sciences, Chinese Academy of Sciences). Briefly, A7r5 cells were cultured with Dulbecco’s modified Eagle’s medium (DMEM, Biological Industries) containing 10 % fetal bovine serum (Biological Industries) at 37 °C in a 5 % CO_2_ chamber. The cells (passages 4–10) were seeded on 96-well plates for 24 h prior to experiments and were then incubated with pu-erh aqueous extract or nifedipine (an inhibitor of voltage-dependent calcium channels) for an additional 24 h. Then, a MTS cytotoxicity assay was performed. Based on the results of the MTS assay (**Supplemental Figure 2**), pu-erh aqueous extract (100 µg/ml), and nifedipine (1 µmol/L) were chosen for the subsequent experiments. Before measuring [Ca^2+^]_i_, A7r5 cells were incubated with 1 µmol/L Fluo-4-AM (Thermo Fisher Scientific) for 30 min in Krebs solution containing 2 mmol/L CaCl_2_ at room temperature. Fluorescent changes in Fluo 4-AM-loaded cells were detected by a Cellomics ArrayScan VTI HCS Reader (Thermo Fisher Scientific) at an excitation wavelength of 488 nm and an emission wavelength of 530 nm on an inverted microscope with a 10 × objective. To elucidate whether pu-erh aqueous extract or nifedipine affected the [Ca^2+^]_i_ level of cells under resting conditions, the fluorescent intensity was recorded before (F_0_) and after (F_1_) the addition of pu-erh aqueous extract or nifedipine. After preincubation with pu-erh aqueous extract or nifedipine for 5 min, KCl (60 mmol/L) was added to induce Ca^2+^ influx, and the fluorescent intensity was recorded (F_2_). Measurements were made at 2 s intervals for 300 s. The relative [Ca^2+^]_i_ level was represented by the ratio of the maximum fluorescent intensity of Fluo-4-AM to the baseline fluorescent intensity of Fluo-4-AM (F_max_/F_0_).

### Statistical Analysis

All data are presented as the mean ± SD. Statistical comparisons of 2 groups were performed by *t*-test, and one-way ANOVA followed by Bonferroni’s test was performed to compare more than 2 groups using SPSS 20.0. A *P* value of less than or equal to 0.05 was regarded as statistically significant. The data were analyzed using GraphPad Prism 5.0 and SPSS 20.0.

## Results

### Effect of Pu-Erh Aqueous Extract on PE- or KCl-Induced Vasoconstriction

**Figure 1:**
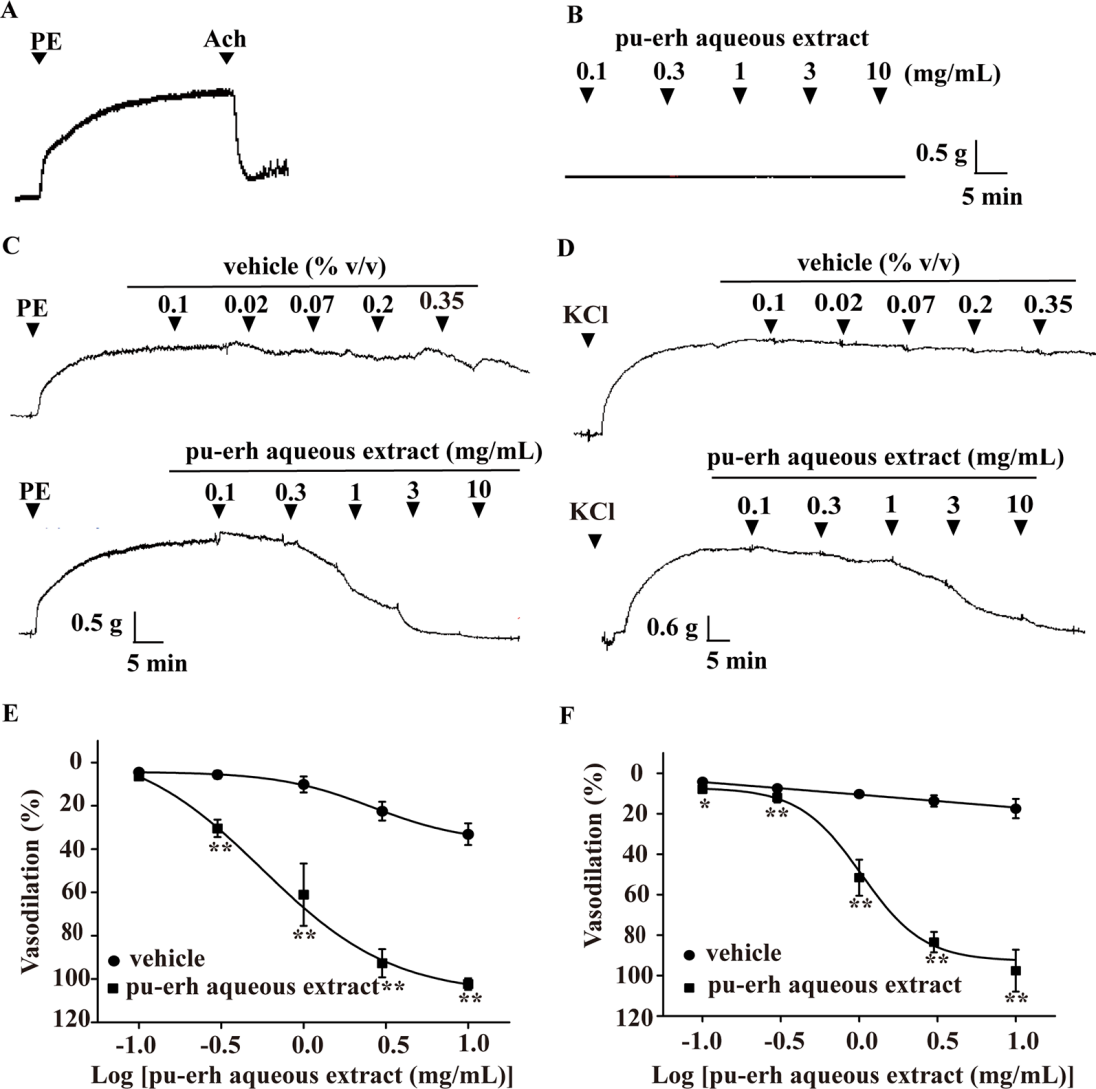
Vasodilation effect of pu-erh aqueous extract on rat thoracic aorta precontracted with PE or KCl. **(A)** Representative trace of vascular tension in an endothelium-intact thoracic aorta. **(B)** Representative trace of vascular tension shows that pu-erh aqueous extract (0.1–10 mg/ml) had no obvious effect on arteries at baseline tension. **(C** and **E)** Pu-erh aqueous extract (0.1–10 mg/ml) concentration-dependently vasodilated endothelium-intact arteries precontracted with PE (1 μmol/L). The vehicle used to dissolve pu-erh aqueous extract was added cumulatively at the same concentration (% v/v). The relaxation ratio is expressed as a percentage of the maximal contraction tension induced by PE or KCl. **(D** and **F)** Pu-erh aqueous extract (0.1–10 mg/ml) concentration-dependently vasodilated endothelium-intact arteries precontracted with KCl (60 mmol/L). n = 6, *P < 0.05, **P < 0.01 vs the vehicle group. PE, phenylephrine; Ach, acetylcholine; KCl, potassium chloride.

In rat thoracic arteries with intact endothelium (**Figure 1A**), pu-erh aqueous extract (0.1–10 mg/ml) did not affect the baseline tension of arteries (**Figure 1B**). However, pu-erh aqueous extract (0.1–10 mg/ml) vasodilated aortic rings precontracted with PE in a concentration-dependent manner (EC_50_, 0.56 mg/ml) (**Figures 1C, E**). Pu-erh aqueous extract was also observed to have a vasodilation effect on KCl-precontracted rings (EC_50_, 1.04 mg/ml) (**Figures 1D, F**).

### Effect of the Endothelium on Pu-Erh Aqueous Extract-Induced Vasodilation

**Figure 2:**
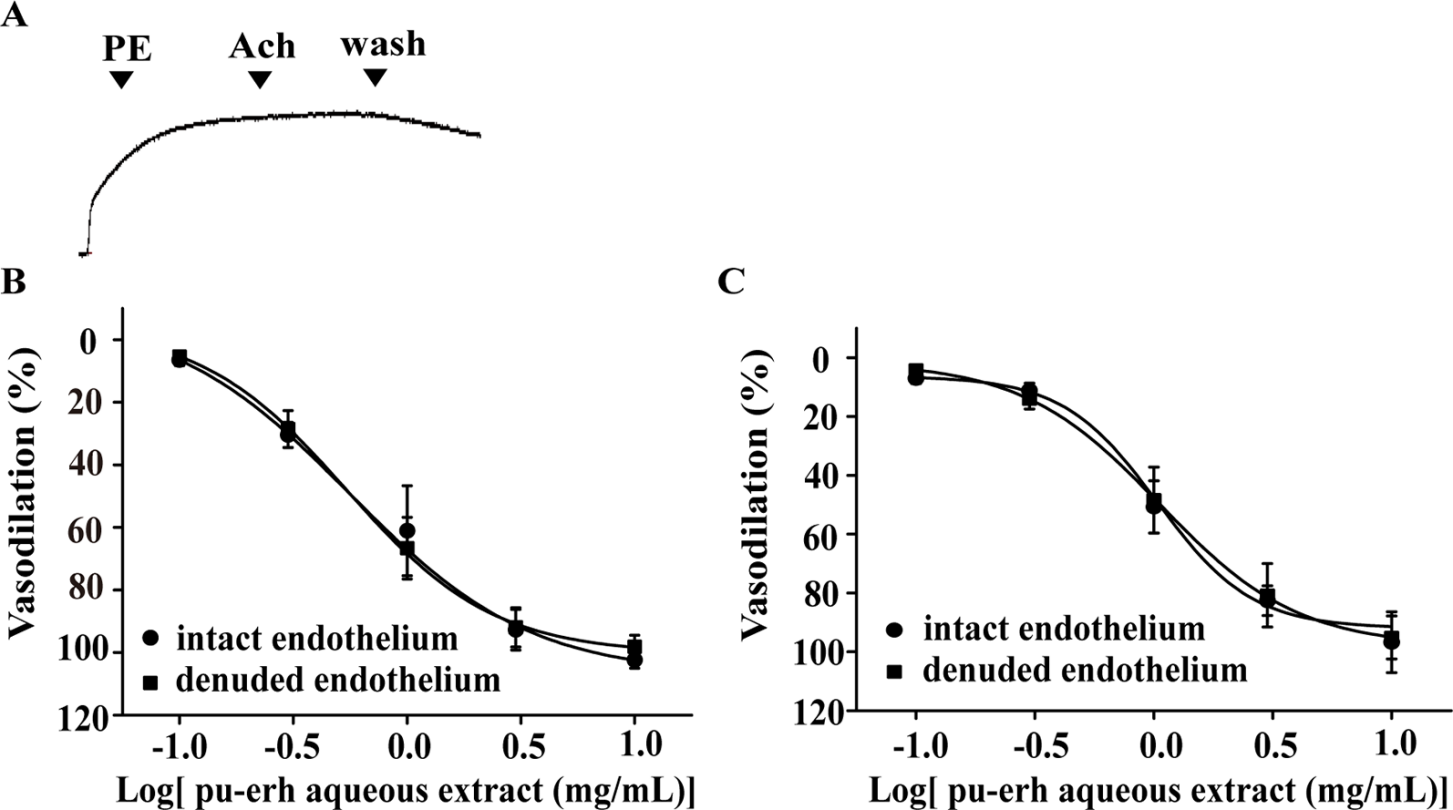
The role of endothelium in the vasodilation effect of pu-erh aqueous extract on rat thoracic aortas precontracted with PE or KCl. **(A)** Representative trace of the vascular tension of a endothelium-denuded thoracic aorta. **(B** and **C)** Dose response curve showing that pu-erh aqueous extract (0.1–10 mg/ml) concentration-dependently vasodilated arteries precontracted with PE (1 μmol/L) **(B)** or KCl (60 mmol/L) **(C)** in an endothelium-independent manner. n = 6. PE, phenylephrine; Ach, acetylcholine.

The endothelium was mechanically removed and endothelium denudation was confirmed by the absence of acetylcholine-induced relaxation (**Figure 2A**). The effect of pu-erh aqueous extract (0.1–10 mg/ml) on the vasodilation of endothelium-denuded arteries (EC50, 0.54 mg/ml) and endothelium-intact arteries (EC50, 0.56 mg/ml) was not significantly different (**Figure 2B**). A similar pattern was also observed in the vasodilation effect of pu-erh aqueous extract on KCl-precontracted arteries with endothelium (EC50, 1.06 mg/ml) and without endothelium (EC50, 1.04 mg/ml) (**Figure 2C**). These results indicated that the vasodilation effect of pu-erh aqueous extract was independent of the endothelium.

### Effect of K^+^ Channels on Pu-Erh Aqueous Extract-Induced Vasodilation

**Figure 3:**
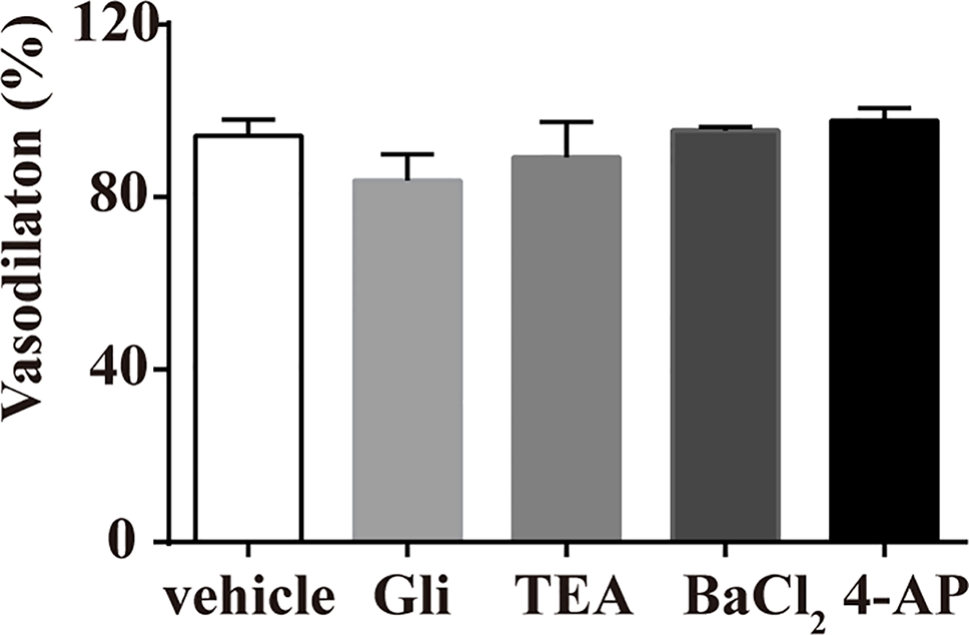
Effects of preincubation with Gli, TEA, BaCl_2_ or 4-AP on the vasodilative effects of pu-erh aqueous extract. Various potassium channel blockers did not inhibit the vasodilative effects of pu-erh aqueous extract (3 mg/ml) on rat thoracic aortas precontracted with PE (1 μmol/L). GLi, glibenclamide; TEA, tetraethylammonium; BaCl_2_, barium chloride; 4-AP, 4-aminopyrimide; KCl, potassium chloride.

Various K^+^ channel blockers were added to aortic rings without endothelium. As shown in **Figure 3**, the vasodilation effect of pu-erh aqueous extract (3 mg/ml) on PE-precontracted rings was not significantly different in the absence or presence of K^+^ channel blockers, including GLi, TEA, BaCl_2_ or 4-AP, indicating that the opening of K^+^ channels might not directly contribute to the mechanism of action of pu-erh tea in vasodilation.

### Effect of Pu-Erh Aqueous Extract on the Vasoconstriction Induced by PE Under Ca^2+^-Free Conditions or Extracellular Ca^2+^ Influx

**Figure 4:**
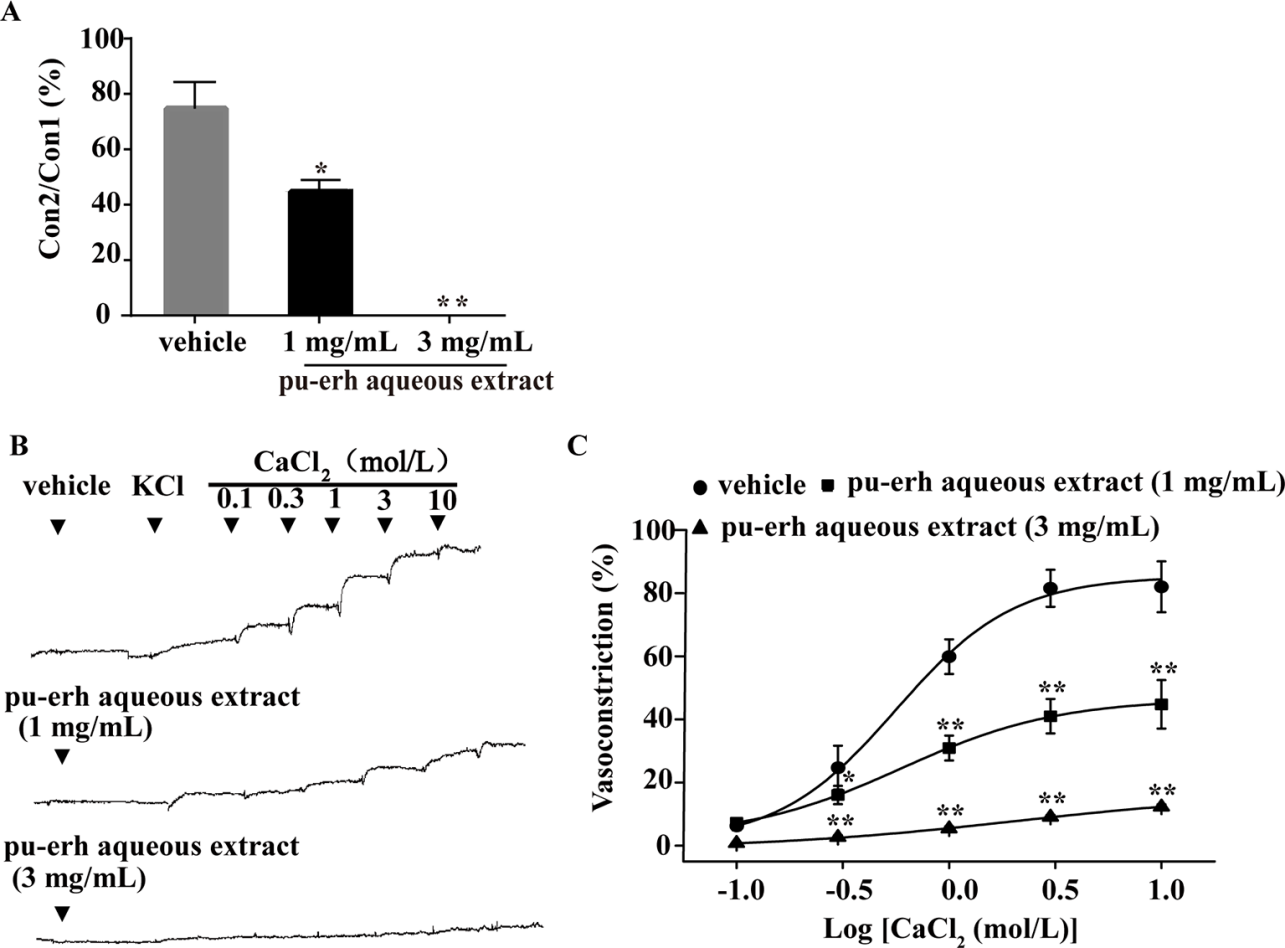
The effect of pu-erh aqueous extract on the contractile response to intracellular Ca^2+^ release or extracellular Ca^2+^ influx. **(A)** Compared with the vehicle, pretreatment with pu-erh aqueous extract (3 mg/ml or 1 mg/ml) reduced the contractile responses of arteries induced by PE-mediated intracellular Ca^2+^ release. Values represent the ratio of the maximum tension of the second constriction induced by PE (Con2) to the first constriction (Con1). **(B)** Representative traces show that pu-erh aqueous extract (3 mg/ml or 1 mg/ml) reduced the contractile response of arteries induced by CaCl_2_ (0.1 mol/L to 10 mol/L). **(C)** Dose response curve of the vasoconstrictive effect induced by extracellular Ca^2+^ influx under depolarizing conditions (60 mmol/L KCl). The vasoconstriction effect was expressed as the percentage of the maximal vasoconstriction induced by KCl (60 mmol/L) in a normal Krebs buffer. n = 5. *P < 0.05, **P < 0.01 vs the vehicle group.

The endothelium-denuded rings were incubated with pu-erh aqueous extract under Ca^2+^-free conditions before PE treatment. As depicted in **Figure 4A**, compared to the vehicle, pu-erh aqueous extract (1 mg/L or 3 mg/L) significantly reduced or even abolished PE-induced vasoconstriction. KCl was applied to endothelium-denuded aortic rings under Ca^2+^-free conditions, followed by the cumulative addition of CaCl_2_ (0.1–10 mol/L) (**Figure 4B**). Compared with the vehicle, pu-erh aqueous extract (1 mg/L or 3 mg/L) substantially reduced the vasoconstriction of arteries induced by increasing concentrations of CaCl_2_ (**Figure 4C**).

### Effect of Pu-Erh Aqueous Extract on [Ca^2+^]i in A7r5 Cells

**Figure 5:**
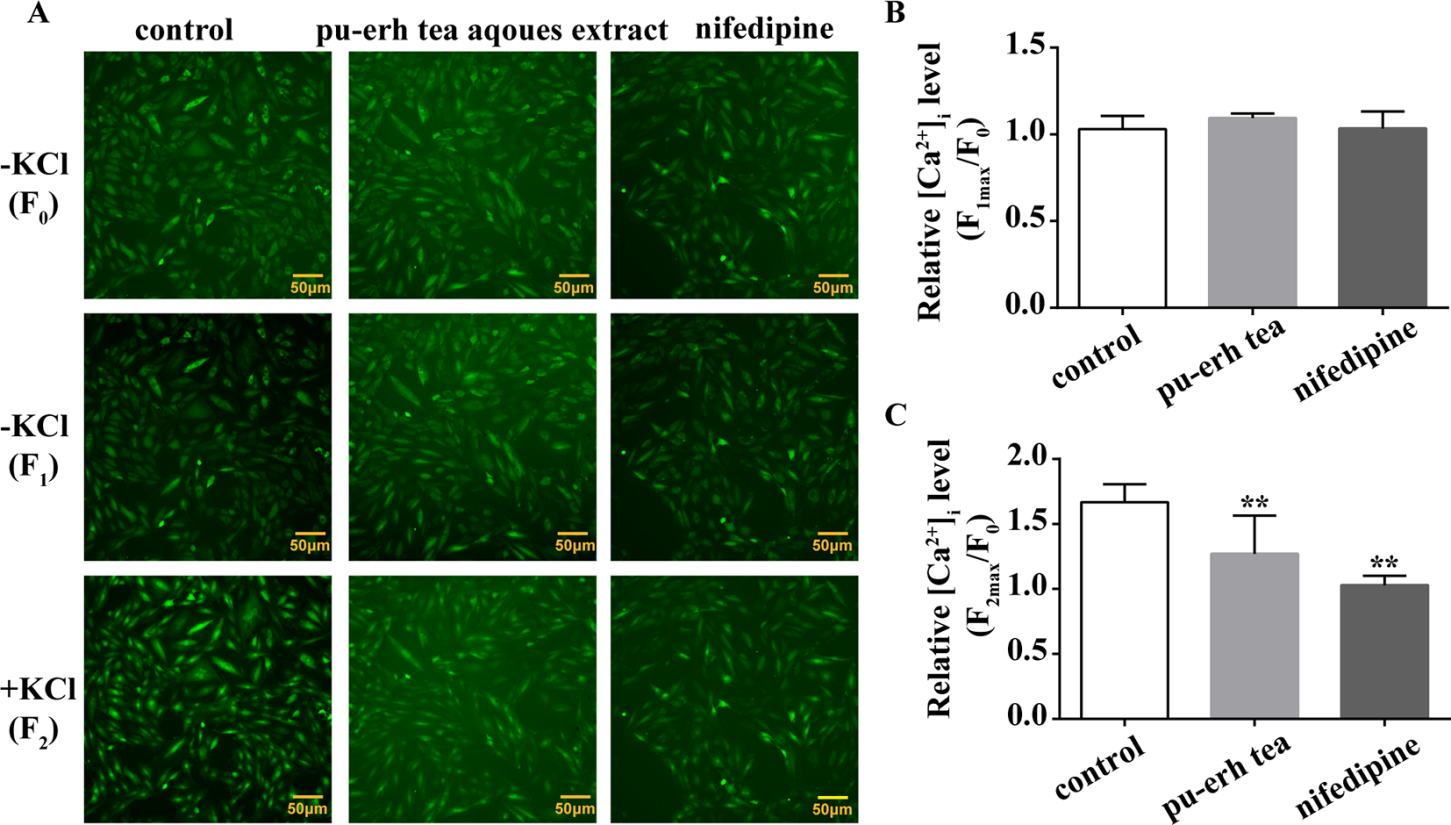
Effect of pu-erh aqueous extract on intracellular calcium in cultured rat aortic A7r5 smooth muscle cells. **(A)** Representative images of calcium imaging. The changes in intracellular calcium ([Ca^2+^]_i_) were assayed by Fluo 4-AM using a Cellomics ArrayScan VTI HCS Reader with an inverted microscope equipped with a 10 × objective. The fluorescent intensity was recorded before (F_0_) and after the addition of 100 μg/ml pu-erh aqueous extract or 1 μmol/L nifedipine (F_1_). Following preincubation with pu-erh aqueous extract or nifedipine, KCl (60 mmol/L)-induced increases in fluorescence (F_2_) were recorded. **(B)** The effect of pu-erh aqueous extract or nifedipine on baseline [Ca^2+^]_i_. The relative [Ca^2+^]_i_ level is represented by the ratio of the maximum fluorescent intensity of Fluo 4-AM to the baseline intensity (F_max_/F_0_). **(C)** The effect of pu-erh aqueous extract or nifedipine on KCl-induced increases in [Ca^2+^]_i_. n = 6. **P < 0.01 vs. the control group.

To further elucidate the impact of pu-erh aqueous extract on [Ca^2+^]_i_, the changes in fluorescent intensity were recorded, and nifedipine was used as a positive control. As demonstrated in **Figure 5**, pu-erh aqueous extract or nifedipine did not obviously influence baseline [Ca^2+^]_i_ levels in A7r5 cells (**Figure 5B**). After the addition of KCl (60 mmol/L), the fluorescence intensity increased, and F_2max_/F_0_ increased by 1.67 ± 0.14 times, suggesting that KCl significantly increased cytosolic [Ca^2+^]_i_. Pu-erh tea aqueous extract and nifedipine obviously decreased F_max2_/F_0_ from 1.67 ± 0.14 to 1.27 ± 0.29 and 1.03 ± 0.07, respectively (**Figure 5C**). These results indicated that pu-erh aqueous extract could reduce cytosolic [Ca^2+^]_i_.

### The Content of Major Components in the Aqueous Extract, Ethanol Extract, Ethanol Precipitate and Chloroform Precipitate of Pu-Erh

As shown in **Table 1**, GA, CAF, TFs, TRs, and TBs accounted for the majority, approximately 60 %, of the pu-erh aqueous extract. CAF accounted for 47.95 % of the ethanol extract, and the content of tea catechins was increased in the ethanol extract compared with the aqueous extract of pu-erh. After the ethanol precipitate was treated with chloroform, the content of CAF decreased from 5.71 % to 0.97 %. In addition, the HPLC profiles of tea catechins, GA, and CAF in the various extracts are shown (**Supplemental Figure 1**)

**Table 1:** The content of catechins, GA, CAF, TBs, TRs and TFs (% w/w).

Component	Pu-erh aqueous extract	Ethanol extract	Ethanol precipitate	Chloroform precipitate	Chloroform extract
GA	1.86	6.3	1.25	0.29	—
CAF	9.02	47.95	5.71	0.97	24.32
EGC	0.10	0.18	—	—	—
C	0.16	0.74	0.11	0.02	—
EC	0.21	0.49	0.17	0.03	—
ECG	0.14	0.15	0.14	0.009	—
EGCG	0.07	0.12	0.06	0.01	—
TBs	39.17 ± 2.20	0.69 ± 0.02	44.84 ± 1.04	41.91 ± 1.09	—
TRs	8.71 ± 1.85	21.86 ± 3.43	4.01 ± 1.38	4.74 ± 0.74	—
TFs	0.77 ± 0.07	0.56 ± 0.11	0.56 ± 0.03	0.50 ± 0.03	—

—, undetected; GA, gallic acid; CAF, caffeine; EGC, epigallocatechin; C, catechin; EC, epicatechin; ECG, epicatechin gallate; EGCG, epigallocatechin gallate; TBs, theabrownins; TRs, thearubigins; TFs, theaflavins.

### Effect of Ethanol Extract, Ethanol Precipitate and Chloroform Precipitate on PE- or KCl-Induced Vasoconstriction

**Figure 6:**
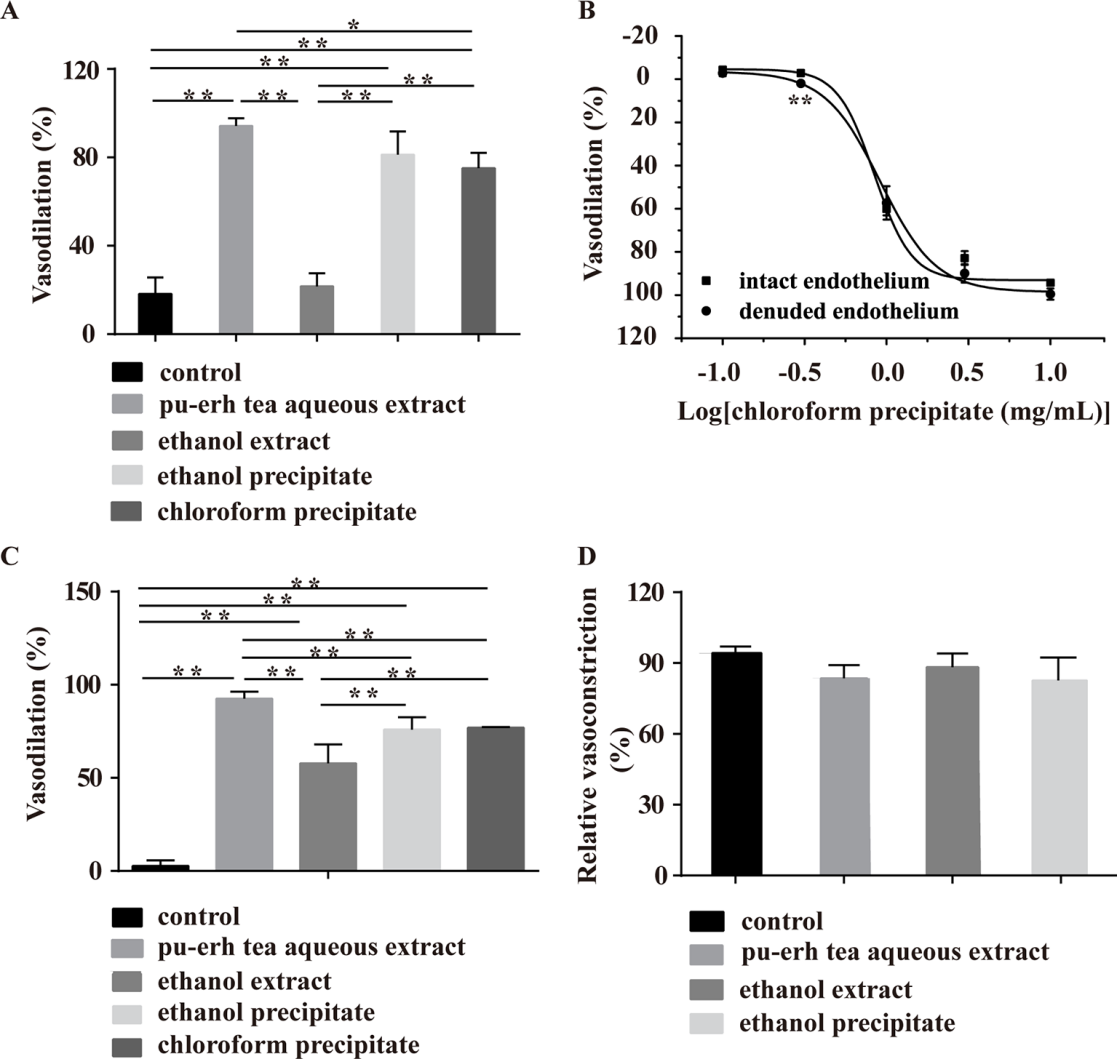
Effect of various fractions of pu-erh aqueous extract on arteries precontracted with PE or KCl. **(A)** The column diagram shows that the ethanol precipitate (5 mg/ml), chloroform precipitate (4.75 mg/ml) and pu-erh aqueous extract (10 mg/ml) but not the ethanol extract (0.7 mg/ml) could vasodilate arteries precontracted with PE. *P < 0.05, **P < 0.01 compared with the control, n = 5. The doses of various fractions were based on the concentration of pu-erh aqueous extract required for the maximum vasodilation effect and the yield of the various extracts. **(B)** The dose response curve shows that the chloroform precipitate (0.1–10 mg/ml) was an endothelium-independent vasodilator. n = 4–6, **P < 0.01 vs the intact endothelium group. **(C)** Ethanol extract (0.7 mg/ml), ethanol precipitate (5 mg/ml), chloroform precipitate (4.75 mg/ml) and pu-erh aqueous extract (10 mg/ml) could relax arteries precontracted with KCl. **P < 0.01 compared with the control, n = 5. **(D)** Compared with the control, pu-erh aqueous extract (10 mg/ml), ethanol extract (0.7 mg/ml), and ethanol precipitate (5 mg/ml) did not reduce the relative ratio of KCl-induced vasoconstriction. n = 5.

The PE- and KCl-induced vasoconstriction models were further employed to screen the main vasoactive components of pu-erh tea. In the PE-induced vasoconstriction model, the ethanol precipitate (5 mg/ml) and chloroform precipitate (4.75 mg/ml), but not the ethanol extract (0.7 mg/ml), showed similar vasodilation effects, but the extent of chloroform precipitate-induced vasodilation was considerably less than that of the pu-erh aqueous extract (10 mg/ml), indicating that the chloroform precipitate and CAF might be the main active fractions (**Figure 6A**). The chloroform precipitate dose-dependently vasodilated arteries with intact endothelium or denuded endothelium (**Figure 6B**), suggesting that the vasodilation effect was endothelium independent.

In the KCl-induced vasoconstriction model, the ethanol extract, ethanol precipitate, and chloroform precipitate induced the vasodilation of arteries, but the activities of these fractions were weaker than that of the aqueous extract. To evaluate whether pu-erh tea extracts exerted vasodilation effects by irreversibly impairing arteries, KCl was employed to induce vasoconstriction after the aortic rings were treated with pu-erh tea extracts and washed repeatedly. The results showed that KCl could induce a normal vasoconstriction response (**Figure 6D**), indicating that these fractions at these concentrations did not influence the basic function of arteries.

### Effect of Catechins, GA, and CAF on PE-Induced Vasoconstriction

**Figure 7:**
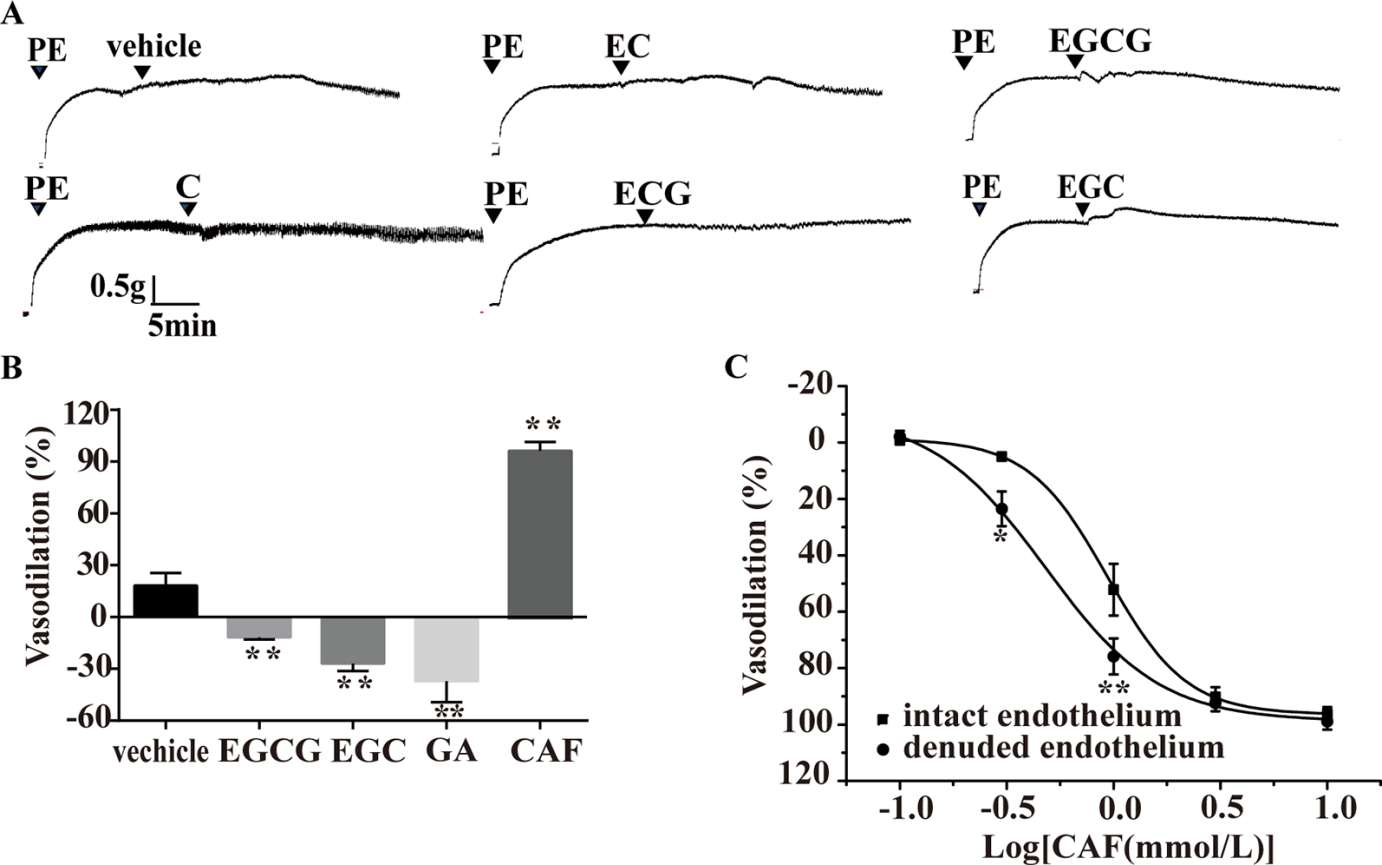
Effect of catechins, GA or CAF on arteries precontracted with PE. **(A)** Representative traces of vascular tension show that C (16.7 μmol/L), EC (9.58 μmol/L), and ECG (9.56 μmol/L) had no effect on aortas, but EGC (9.91 μmol/L) and EGCG (4.61 μmol/L) induced transient vasoconstriction. **(B)** EGCG (4.61 μmol/L), EGC (9.91 μmol/L), and GA (1 mmol/L) produced vasoconstrictive effects, but CAF (1.39 mmol/L) induced vasodilation, n = 4, **P < 0.01 vs. the vehicle group. The doses of various compounds were based on the concentration of pu-erh aqueous extract required for the maximum vasodilation effect and the content of the compounds. **(C)** The dose response curve of CAF (0.1–10 mmol/L), n = 5, *P < 0.05, **P < 0.01 vs. the intact endothelium group. C, catechin; EC, epicatechin; EGCG, epigallocatechin-3-gallate; ECG, epicatechin-3-gallate; EGC epigallocatechin; GA, gallic acid; CAF; caffeine.

As shown in **Figures 7A, C** (55.1 µmol/L), EC (72.3 µmol/L), and ECG (31.6 µmol/L) had no obvious effect on arteries precontracted with PE. However, compared with the vehicle, EGCG (15.2 µmol/L), EGC (32.7 µmol/L), and GA (1 mmol/L) induced slight vasoconstriction in arteries, while CAF induced obvious vasodilation (**Figure 7B**). CAF dose-dependently vasodilated endothelium-intact arteries precontracted with PE (EC_50_, 0.48 mg/ml) and endothelium-denuded arteries precontracted with PE (EC_50_, 0.94 mg/ml) (**Figure 6C**). As shown in **Figure 6C**, the vasodilation effect of CAF was partly eliminated by the removal of the endothelium.

### Effect of Catechins, GA, and CAF on KCl-Induced Vasoconstriction

**Figure 8:**
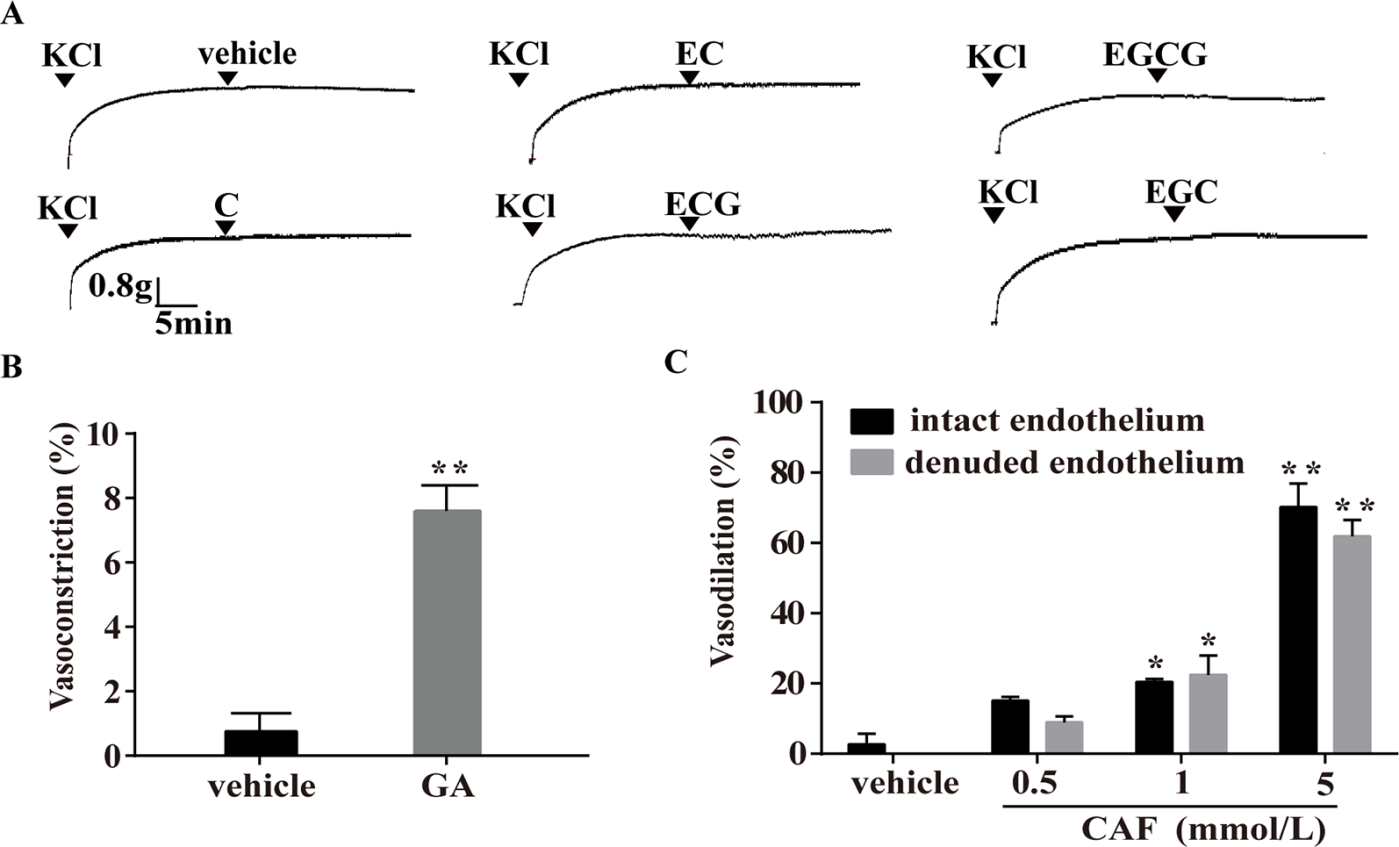
Effect of catechins, GA, or CAF on arteries precontracted with KCl. **(A)** Representative traces show that C, EC, ECG, EGC, and EGCG had no effect on arteries precontracted with KCl. The doses were similar to those used in the PE-induced vasoconstriction model. **(B)** Vasoconstriction effect of GA (1 mmol/L) on arteries precontracted with KCl is shown. The values are expressed as the percentage increase in the maximum constriction induced by KCl. **(C)** CAF (0.5 mmol/L, 1 mmol/L, or 5 mmol/L) relaxed the KCl-induced constriction of endothelium-intact and endothelium-denuded rat thoracic aortas. n = 6. *P < 0.01, **P < 0.01 vs. the vehicle group. EC, epicatechin; EGCG, epigallocatechin-3-gallate; C, catechin; ECG, epicatechin-3-gallate; EGC, epigallocatechin; GA, gallic acid; CAF; caffeine.

In the KCl-induced vasoconstriction model, as depicted in **Figure 8**, compared with the vehicle, C, EC, ECG, EGCG, and EGC showed no obvious effects on arteries (**Figure 8A**), and GA induced slight vasoconstriction (**Figure 8B**). However, at the concentration of 0.5 mmol/L, 1 mmol/L, or 5 mmol/L, CAF induced significant vasodilation in endothelium-intact arteries, and this effect was not eliminated by the removal of the endothelium (**Figure 8C**).

### Effect of TBs on KCl-Induced Vasoconstriction

**Figure 9:**
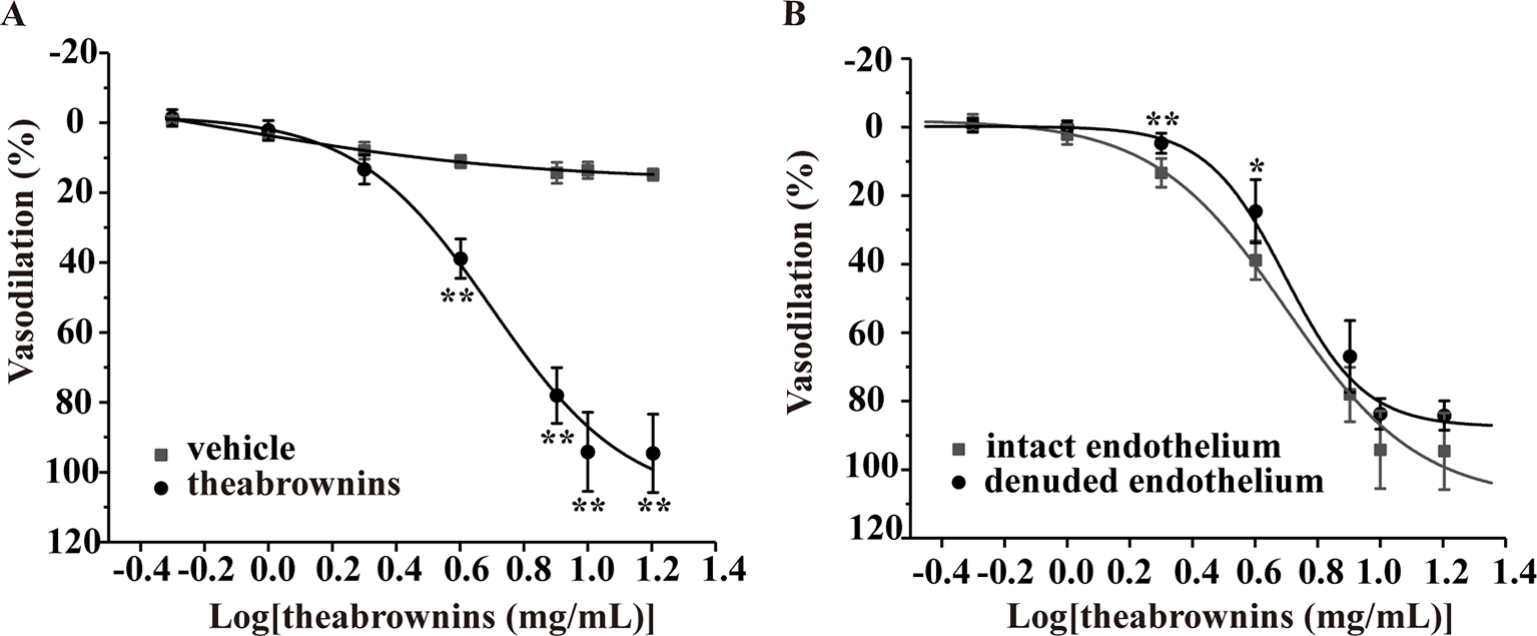
Effect of TBs on rat thoracic aortas precontracted with KCl. **(A)** Compared with the vehicle, TBs (0.5–16 mg/ml) significantly concentration-dependently vasodilated endothelium-intact arteries precontracted with KCl, **P < 0.01, n = 5. **(B)** TBs (0.5–16 mg/ml) vasodilated endothelium-denuded arteries and endothelium-intact arteries precontracted with KCl, and the vasodilation in these two groups was not significantly different, *P < 0.01, **P < 0.01 vs. the intact endothelium group, n = 5. TBs, theabrownins.

The effect of TBs on arteries was evaluated in the KCl-induced vasoconstriction model. As shown in **Figure 9**, compared with the vehicle, TBs concentration-dependently (0.5–16 mg/ml) relaxed arteries precontracted with KCl (EC_50_, 4.7 mg/ml) (**Figure 9A**). The vasodilation effect of TBs in endothelium-intact arteries was not significantly different from that in endothelium-denuded arteries (EC_50_, 5.41 mg/ml) (**Figure 9B**). These results showed that the vasodilation effect of TBs was endothelium independent.

## Discussion

Although pu-erh aqueous extract could lower blood pressure in spontaneous hypertension rats (Li et al., 2012; Li et al., 2015), its action mechanisms remain unclear. Considering that blood pressure-lowering property is closely associated with vasodilation, we investigated the direct effects of pu-erh tea on *ex vivo* arteries, as well as explored the mechanisms and main active components. In this work, it demonstrated that pu-erh aqueous extract vasodilated thoracic arteries of rat in an endothelium-independent manner, and that it might reduce the vessel contractility by inhibiting the influx of extracellular Ca^2+^ to smooth muscle cell. Moreover, CAF and TBs might be the two main active components in pu-erh tea. Our work proposed a potential antihypertensive mechanism of pu-erh aqueous extract and its possible active components. However, due to the complexity of hypertension pathophysiology, other hypotensive mechanisms might be involved and require further research. Nevertheless, the finding that pu-erh tea dilated blood vessels *ex vivo* could provide a clue for the long-term studies on the antihypertensive efficacy of pu-erh tea.

The vascular endothelium synthesizes and releases several vasodilative substances, including nitric oxide (NO), endothelium-derived hyperpolarizing factor (EDHF), and prostacyclin (PGI_2_) (Triggle et al., 2012). However, the vasodilatory effect of pu-erh aqueous extract was found to be independent of vascular endothelium. It indicated that the intracellular signal pathways (PI3K/Akt/NO, COXs/PGI_2_, or EDHF, etc.) associated with endothelium-dependent vasodilation might not directly contribute to the vasodilative mechanism of pu-erh tea.

High concentration of KCl can evoke the depolarization of cell membrane and increase the Ca^2+^ influx through voltage-dependent calcium channels (VDCCs), resulting in vasoconstriction (Mills et al., 2015). In contrast, the opening of potassium channels leads to the hyperpolarization of smooth muscle cells, consequently, the activation of VDCCs is inhibited, and thus vasodilation (Quast et al., 1994). Whereas, the opening of K^+^ channels might not directly contribute to the vasodilation effect of pu-erh aqueous extract. Furthermore, pu-erh aqueous extract reduced the vasoconstriction induced by CaCl_2_ under KCl-depolarizing conditions and attenuated the KCl-induced [Ca^2+^]_i_ increase in A7r5 cells. These results indicated that the reduction of extracellular Ca^2+^ influx by blocking VDCCs might be a main mechanism contributing to the vasodilative effect of pu-erh tea.

PE, an α-adrenergic receptor agonist, can induce vasoconstriction by increasing extracellular Ca^2+^ influx *via* receptor-operated calcium channels (ROCCs), mobilizing Ca^2+^ from intracellular stores *via* inositol-1,4,5-trisphosphate (IP_3_) receptors, and inducing the generation of diacylglycerol which activates protein kinase C (McFadzean and Gibson, 2002). We found that pu-erh aqueous extract inhibited the vasoconstriction induced by PE in Ca^2+^-free buffer, implying that pu-erh tea might inhibit Ca^2+^ release from sarcoplasmic reticulum stores, thus decrease [Ca^2+^]_i_ and relax the aorta.

Previous studies reported that the vascular protection provided by green tea or black tea was due to the regulation of endothelial function by their polyphenol components (Duffy et al., 2001; Sano et al., 2004; Grassi et al., 2009; Lorenz et al., 2009). These findings are inconsistent with the vascular effect of pu-erh aqueous extract may be mainly due to the different degrees of fermentation. The main components of green tea are catechins (Lorenz et al., 2009). In contrast, the content of catechins is quite low in fermented tea due to extensive oxidation. Therefore, a high content of TFs and TRs was detected in black tea (Menet et al., 2004), while TRs and TBs were mainly detected in pu-erh tea (Gong et al., 2010).

EGCG (Potenza et al., 2007; Al Disi et al., 2015; Anwar et al., 2016) and EC (Galleano et al., 2013; Jackson et al., 2018) were reported to lower blood pressure in multiple hypertension models. However, due to the quite low content of these compounds in pu-erh tea and the findings that C, EC, ECG, EGC, EGCG, or GA had no effects on vasodilation, which were consistent to the previous study (Andriambeloson et al., 1998), we speculated that catechins might not be the main components of pu-erh tea in lowing blood pressure. We also found that CAF induced the endothelium-dependent vasodilation of arteries precontracted with PE and the endothelium-independent vasodilation of arteries precontracted with KCl. These different effects might be due to the different vasoconstrictors and various action mechanisms of CAF on the vascular wall, e.g. inhibition of voltage-dependent Ca^2+^ channels (Hughes et al., 1990), inhibition of MLC kinase (Ozaki et al., 1990) and increased “noncontractile” Ca^2+^ (Watanabe et al., 1992). Previously, the effects of CAF in lowing blood pressure were reported, for instance, CAF improved fructose-induced hypertension (Yeh et al., 2014), antagonized salt-sensitive hypertension (Yu et al., 2016; Wei et al., 2018), and prevented diet-induced hypertension in rats (Conde et al., 2012).

In addition, TRs and TFs accounted for about 9.5 % in pu-erh tea aqueous extract. Their endothelial-dependent vasodilation effects were observed in a previous study (Lorenz et al., 2009). Our data showed that TBs was the main component of pu-erh tea aqueous extract with the highest content (40 %) and that TBs vasodilated arteries in an endothelium-independent manner. TBs is reported to exhibit various bioactivities, such as cholesterol-lowering (Gong et al., 2010), prevention of bone loss (Liu et al., 2018), and inducing the apoptosis of human carcinoma cells (Wu et al., 2016; Zhou et al., 2017; Jin et al., 2018b). However, the composition of TBs is complex, which contains multiple polymers with different degrees of polymerization, so we speculated that some of them might exhibit higher activity than TBs in total. The effect of TBs on hypertensive animals has not been reported. Therefore, the chemical composition and structure of TBs, the pharmacokinetics and the *in vivo* hypotensive effect of TBs require further studies.

Taken together, the results suggested that TBs and CAF might be the two main active components contributing to the vasodilative effect of pu-erh tea aqueous extract.

## Conclusion

**Figure 10:**
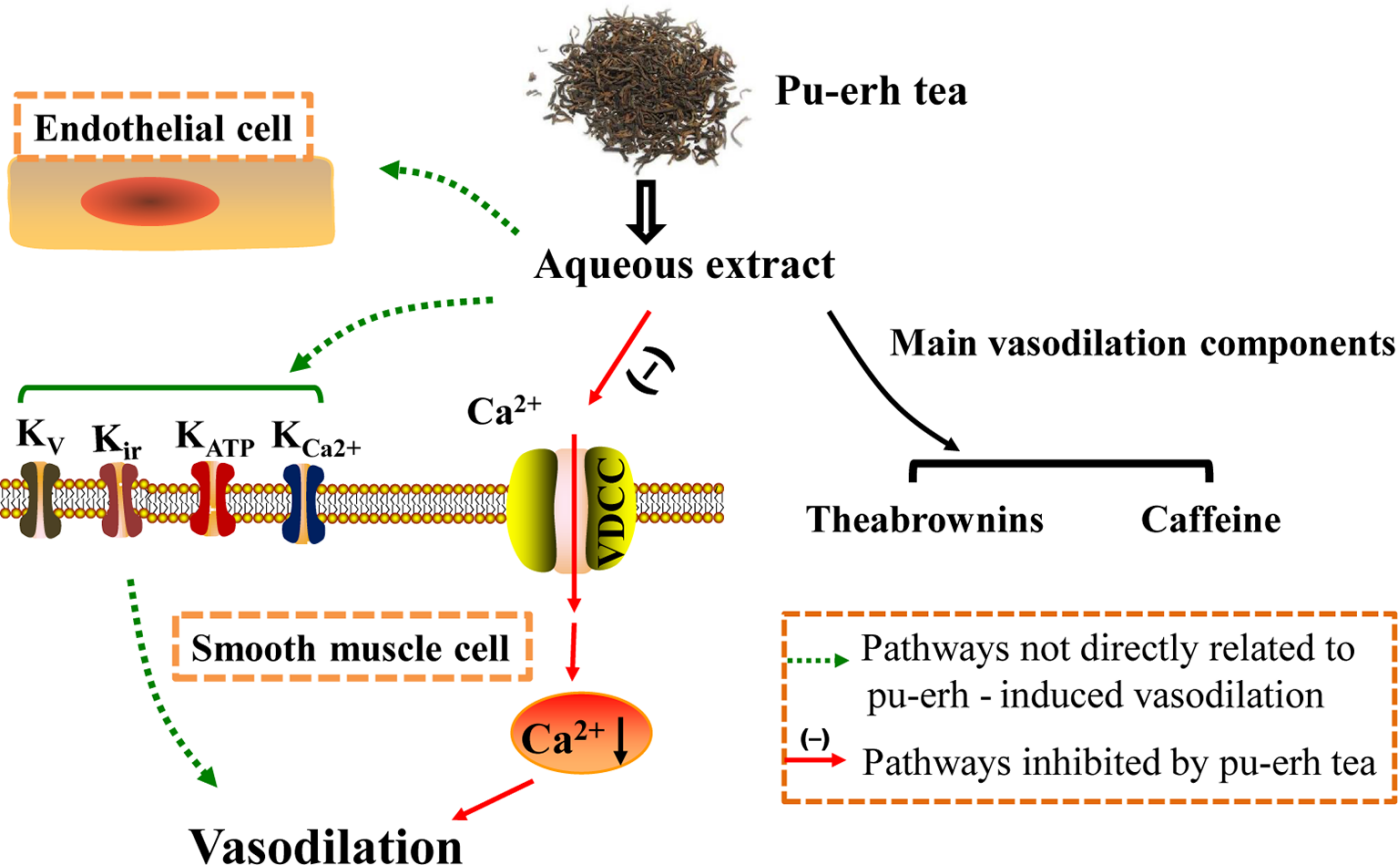
The study schematic representing the mechanism of pu-erh tea-induced vasodilation in the rat thoracic aorta. Pu-erh aqueous extract vasodilated arteries in an endothelium-independent manner, which might partly by inhibiting extracellular Ca2+ influx. Additionally, TBs and CAF should be the main active components. TBs, theabrownins; CAF, caffeine.

Pu-erh aqueous extract and TBs could induce vasodilation in an endothelium-independent manner. Moreover, the vasodilation effect of pu-erh aqueous extract might partly be attributed to the reduction of extracellular Ca^2+^ influx. CAF and TBs might be the two main active components in pu-erh tea (**Figure 10**). Our results improve the understanding of the antihypertensive effect of pu-erh tea and support the health-promoting effects of pu-erh tea as a daily drink.

## Data Availability Statement

The datasets generated for this study are available on request to the corresponding author.

## Ethics Statement

The animal study was reviewed and approved by the Experimental Animal Ethic Committee of Kunming Institute of Botany, Chinese Academy of Sciences.

## Author Contributions

XJ, JZ, and DL conceived and designed the experiments. DL, XC, XZ, and SL carried out the experiments. DL, XC, and XZ analyzed the data. JZ and JL contributed reagents/materials/analysis tools. XJ, JZ, DL, and JX wrote and revised the manuscript. All authors read and approved the final manuscript.

## Conflict of Interest

The authors declare that the research was conducted in the absence of any commercial or financial relationships that could be construed as a potential conflict of interest.

## Supplementary Material

HPLC profiles of tea catechins, GA, and CAF in various extracts are provided in the supplemental materials. The effects of pu-erh aqueous extract on A7r5 cell viability are also shown.
