# Supplementary material for: Pu-Erh Tea Relaxes the Thoracic Aorta of Rats by Reducing Intracellular Calcium
Source: Front Pharmacol. 2019 Nov 28;10:1430. doi: 10.3389/fphar.2019.01430 (PMC6892945; doi:10.3389/fphar.2019.01430)
Supplement: Supplementary file 1 [file DataSheet_1.docx]

Supplementary Material


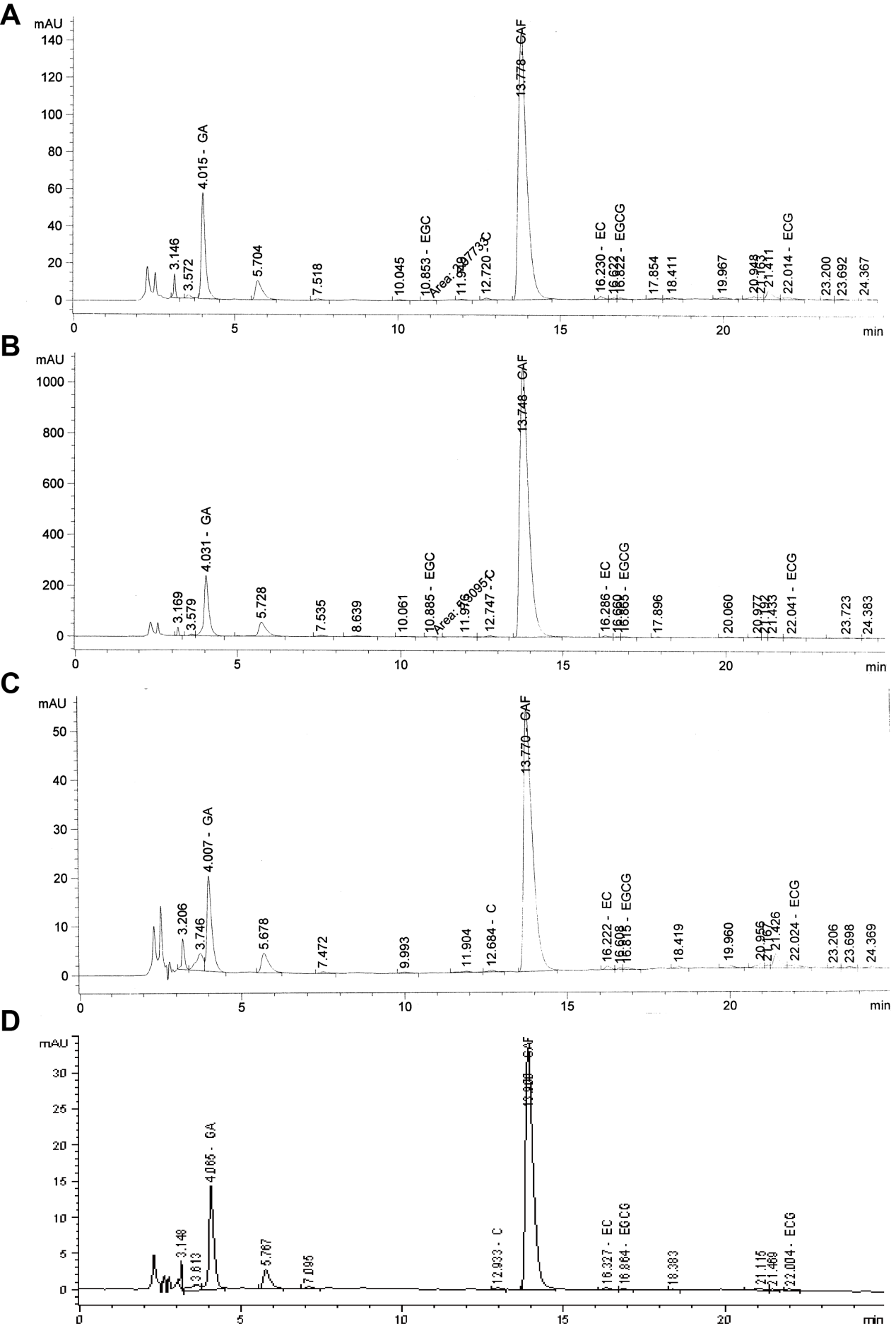


**Supplementary Figure 1** HPLC profiles of tea catechins, GA and CAF in various extracts. HPLC profiles of tea catechins, GA and CAF in the aqueous extract (A), ethanol extract (B), ethanol precipitate (C), and chloroform precipitate (D) of pu-erh tea are shown. The chromatography analysis was performed on an Agilent ZORBAX SB-C18 column (4.6 mm × 250 mm, 5 μm) at 40 ℃. A gradient elution was performed with various proportions of solvent A (acetonitrile) to solvent B (water-formic acid with 0.5 % formic acid), with a flow rate of 1 mL/min and an injection volume of 5 μL. The procedure was performed as follows: 0-5 min, 8 % A, 92 % B; 5-23 min, 8-25 % A, 92-75 % B; and 23-23.01 min, 25-8 % A, 75-92 % B. The acquisition wavelength was 280 nm.


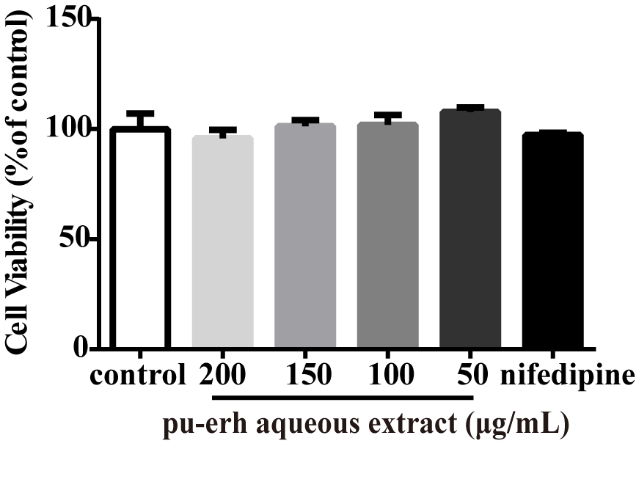


**Supplementary Figure 2** Effect of pu-erh aqueous extract or nifedipine on A7r5 cell viability. Rat thoracic aortic smooth muscle A7r5 cells were seeded on 96-well plates for 24 h and incubated with pu-erh aqueous extract (200 μg/mL, 150 μg/mL, 100 μg/mL, or 50 μg/mL) or nifedipine (1 μmol/L) for an additional 24 h. Then, an MTS assay was performed. The summarized data show that pu-erh aqueous extract (200 μg/mL, 150 μg/mL, 100 μg/mL, or 50 μg/mL) or nifedipine (1 μmol/L) did not obviously affect cell viability.
