# Supplementary figures and images for: Pu-Erh Tea Relaxes the Thoracic Aorta of Rats by Reducing Intracellular Calcium
Source: Front Pharmacol. 2019 Nov 28;10:1430. doi: 10.3389/fphar.2019.01430 (PMC6892945; doi:10.3389/fphar.2019.01430)

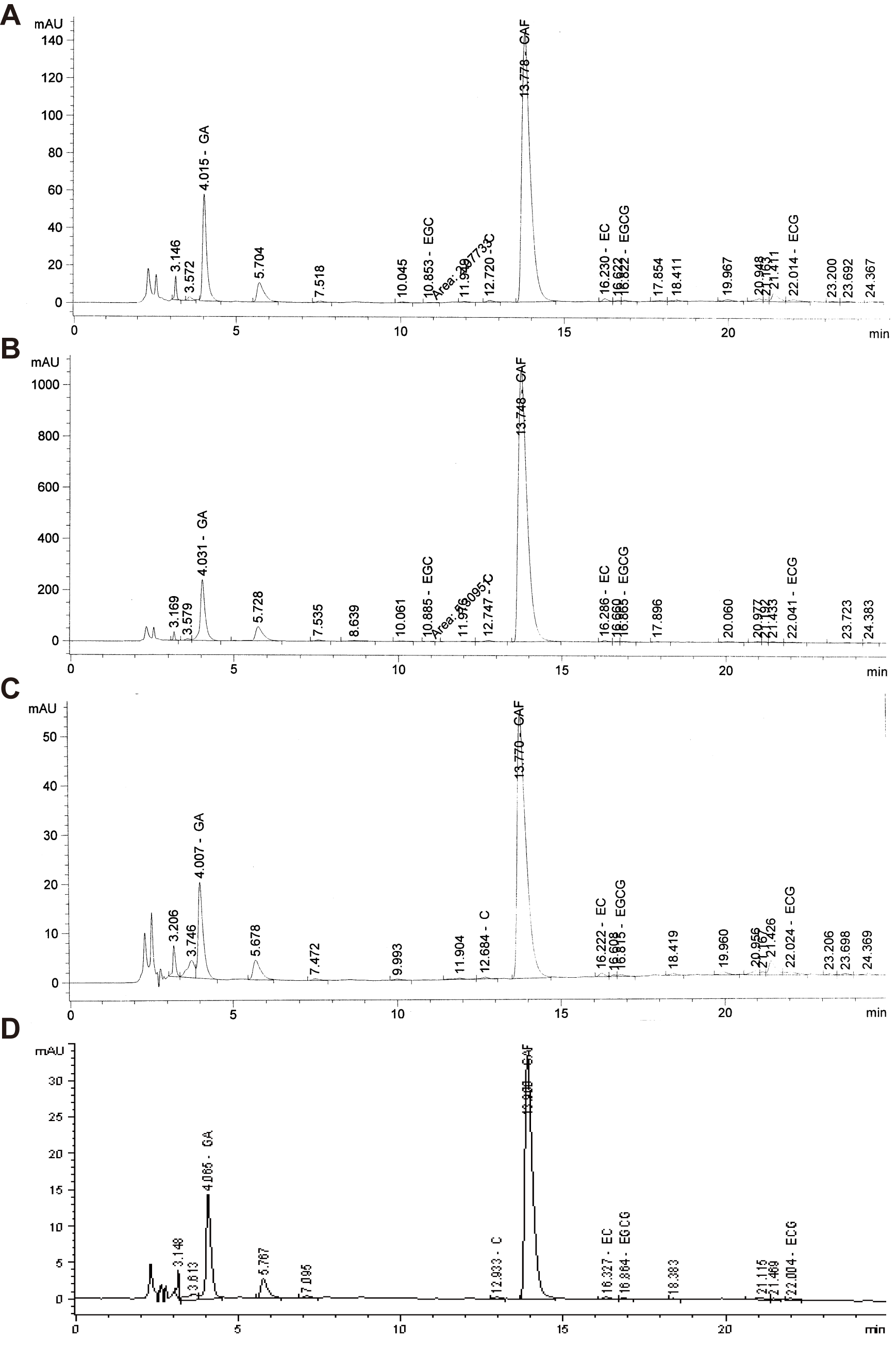

Supplement: Supplementary file 2 [file Image_1.tif]

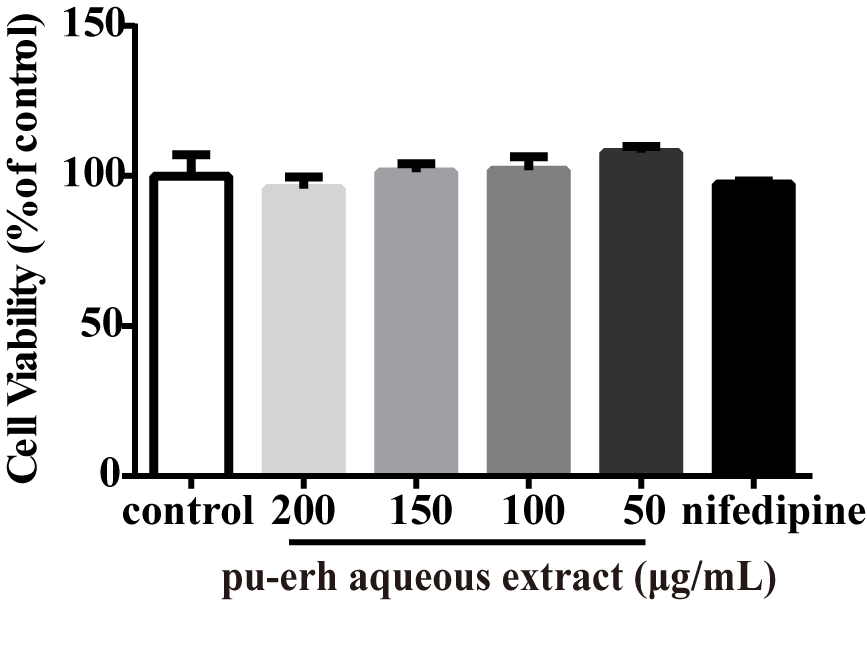

Supplement: Supplementary file 3 [file Image_2.tif]
